# Supplementary material for: Neuromuscular and Neuromuscular Junction Manifestations of the PURA-NDD: A Systematic Review of the Reported Symptoms and Potential Treatment Options
Source: Int J Mol Sci. 2023 Jan 23;24(3):2260. doi: 10.3390/ijms24032260 (PMC9917016; doi:10.3390/ijms24032260)
Supplement: Supplementary file 1 [file ijms-24-02260-s001.zip › ijms-2102345-supplementary.pdf]

Table S1. Studies included in this review (chronological order).

Study	PURA syndrome	5q31.3 microdeletion syndrome	Reference
Hosoki et al. 2012		3	[7]
Brown et al. 2013		2	[40]
Hunt et al. 2014	4		[9]
Lalani et al. 2014	11		[6]
Bonaglia et al. 2015		1	[41]
Tanaka et al. 2015	6		[42]
Okamoto et al. 2017	1		[43]
Rezkalla et al. 2017	1		[44]
Lee et al. 2017	14		[36]
Reijnders et al. 2018	32		[39]
Mayorga et al. 2018	1		[45]
Shimojima et al. 2018		1	[46]
Trau et al. 2019	1		[47]
Rodriguez-Garcia et al. 2020	1		[48]
Jezela Stanek et al. 2020	1		[49]
Clinquina et al. 2021	1		[50]
Mroczek et al. 2021	1		[51]
Liu et al. 2021	1		[52]
Solazzi et al. 2021	1		[53]
Choi et al. 2021	5	2	[54]
Lin et al. 2021	1		[55]
Johannesen et al. 2021	67		[35]
Wyrebek et al. 2022	1		[34]
Nogueira et al. 2022	1		[56]
Fukuda et al. 2022	1		[57]
Qashquari et al.2022	1		[38]
Dai et al.2022	24	1	[8]
